# Case Report: pembrolizumab monotherapy achieves durable disease control in a patient with *BRAF* V600E-mutant advanced lung adenocarcinoma and high PD-L1 expression intolerant to *BRAF*/*MEK* inhibition

**DOI:** 10.3389/fonc.2026.1720971

**Published:** 2026-02-16

**Authors:** Wei Zhao, Rong Zhou, Chunlai Feng

**Affiliations:** Department of Respiratory and Critical Care Medicine, The Third Affiliated Hospital of Soochow University, Changzhou, China

**Keywords:** *BRAF* V600E mutation, case report, immune checkpoint inhibitor, non-small cell lung cancer, PD-L1

## Abstract

**Purpose:**

To investigate the follow-up treatment options and prognosis in patient with non-small cell lung cancer (NSCLC) harboring a *BRAF* V600E mutation who is intolerant to targeted therapy.

**Case presentation:**

A patient with a 30-year smoking history and well-controlled hypertension developed lung adenocarcinoma (left upper lobe; T2aN2MO, Stage IIIA; Karnofsky Performance Status: 90) after radical lung cancer surgery. The patient was later hospitalized for “recurrent hemoptysis” and received pemetrexed adjuvant chemotherapy combined with cisplatin. The patient, subsequently developed focal brain metastasis for which he received radiotherapy. However, the patient experienced recurrent pulmonary lesions. The genetic testing and immunohistochemistry analysis revealed the presence of a *BRAF* V600E mutation and high PD-L1 expression. Consequently, a targeted therapy regimen combining low to moderate doses of dabrafenib and trametinib was initiated. However, the patient developed severe and rare adverse reactions, prompting a switch to pembrolizumab monotherapy.

**Outcomes:**

The patient exhibited significant symptomatic improvement, with a marked reduction in recurrent and metastatic lesions. Subsequent follow-up assessments indicated stable disease with no evidence of progression. Moreover, only one low-grade immune-related adverse event was observed during the course of immunotherapy.

**Conclusion:**

Patients with advanced NSCLC and *BRAF* mutations who cannot tolerate targeted therapy are expected to benefit from immunotherapy.

## 1 Introduction

Currently known driver genes in non-small cell lung cancer (NSCLC) include *EGFR*, *HER2*, *KRAS*, *ROS1*, *ALK*, *MET*, *BRAF*, *RET, NTRK and NRG1*. In addition, there are some co-mutated genes (e.g., *TP53*, *STK11*, *KEAP1*) that, while non-targetable, have important biological significance. The detection rate of V-Raf murine sarcoma viral oncogene homolog B1 in NSCLC is 1.5–3.5% (1), and these mutations are associated with poor prognosis of NSCLC (2).

Over the past several years, the U.S. Food and Drug Administration (FDA) has approved combinations of *BRAF* and *MEK* inhibitors for the treatment of *BRAF* V600E-mutant advanced NSCLC, including dabrafenib (*BRAF* inhibitor) in combination with trametinib (*MEK* inhibitor) in 2017, and more recently (2023) encorafenib (*BRAF* inhibitor) in combination with binimetinib (*MEK* inhibitor). According to the current guidelines, targeted therapy with *BRAF* in combination with *MEK* inhibitors has become the first-line treatment for patients with *BRAF* V600E-mutated advanced NSCLC. However, with the wide application of dual-targeted drugs, some patients are struggling to tolerate this treatment because of serious and rare adverse effects of the targeted drugs.

In the present case, following dual-agent combination targeted therapy, the patient with *BRAF* V600E mutant recurrent metastatic NSCLC experienced serious adverse reactions, including high-grade fever, rhabdomyolysis, abnormal liver function, and abnormal renal function. Pembrolizumab monotherapy, initiated afterward, demonstrated a favorable and long-lasting response for at least 54 months.

## 2 Case presentation

A 67-year-old male with a history of hypertension, well-controlled with the regular use of Irbesartan Hydrochlorothiazide tablets, and a 30-year smoking history (averaging 20 cigarettes/day), was admitted on November 8, 2019, for recurrent hemoptysis lasting over 1 month. Initial chest computed tomography (CT) revealed a left upper lobe lesion suspicious for malignancy. Tumor markers were as follows: neuron-specific enolase (NSE), 18.55 ng/mL; carcinoembryonic antigen (CEA), 12.94 ng/mL; and pro-gastrin-releasing peptide (ProGRP), 77.46 pg/mL. Furthermore, positron emission tomography/CT (PET/CT) revealed a hypermetabolic lesion in the posterior segment of the right upper lung apex mass and a ground-glass opacity suspicious for malignancy, along with abnormal fludeoxyglucose (FDG) uptake in the aortic window and para-aortic arch enlarged lymph nodes.

On December 24, 2019, a percutaneous lung biopsy of the lesion showed a small amount of lung tissue with interstitial fibrosis and mucoid degeneration, along with individual hyperplastic glands and atypical cells. Despite the lack of definitive tumor evidence from the biopsy, persistent hemoptysis and PET/CT findings raised the suspicion for malignancy. Afterward, a thoracoscopic left upper lobectomy was performed for hemostasis. Intraoperative frozen section pathology confirmed poorly differentiated NSCLC, prompting radical surgery (thoracoscopic left upper lobectomy, hilar and mediastinal lymph node dissection). Postoperative pathological results indicated poorly differentiated infiltrating adenocarcinoma in the left upper lobe, predominantly solid type (80%), with a sarcomatoid carcinoma (10%), and acinar predominant type (10%) components. The tumor size was 3.5 × 3 cm, with infiltration of the pleura (+). The bronchial resection margin was clear for cancer and had no sign of cancer metastasis in the examined bronchial lymph nodes (0/3). However, mediastinal lymph nodes demonstrated cancer metastasis (Group 5: 2/2, Group 10: 2/4). Lymph nodes in Group 11 showed reactive hyperplasia (0/3). While immunohistochemical results were negative for CgA, Syn, CD56, NapsinA, CK 5/6, P63, and P40, they were positive for TTF-1, CK7, Ki67 (60%), and AE1/AE3. Assessment of PD-L1 status using the Dako 22C3 antibody revealed a high tumor proportion score (TPS) of ≥60%. Postoperatively, the patient was diagnosed with lung adenocarcinoma (upper left lung, T2aN2M0, stage IIIA; Karnofsky Performance Status, 90 points).From February to April 2020, four cycles of systemic chemotherapy with pemetrexed (0.8 g) and cisplatin (100 mg) were administered. Post-chemotherapy evaluation confirmed stable disease with no metastasis.

On July 6, 2020, the patient was hospitalized for a follow-up examination. The chest CT indicated an increase in the left pleural effusion and left periaortic pulmonary hilar lymph nodes. To assess for systemic metastasis, a follow-up PET/CT was performed after left lung cancer surgery. Multiple FDG metabolic foci were observed, suggesting lymph node metastases in the bilateral supraclavicular region (particularly on the left side), main pulmonary artery window, aortic arch area, and left pulmonary hilum. Localized FDG metabolic increase was noted in the left lung apex pleura, indicating pleural metastasis. Additionally, a left cerebellar nodule with FDG metabolic increase was noted, suggesting a possibility of metastasis. Compared to the PET/CT from December 19, 2019, all of the aforementioned lesions were considered new metastatic foci. Further cranial enhanced magnetic resonance imaging (MRI) showed an enhancement indicating a newly developed mass (since the previous scan on 26th March 2020) in the left cerebellar hemisphere (approximately 1.6 cm in diameter), suggesting a potential metastatic lesion. Bronchoscopy revealed a narrowing of the external compression at the opening of the basal segment of the right lower lobe, with the distal lumen remaining clear. Endobronchial ultrasound (EBUS) examination depicted a low echo mass measuring 1.1 × 1.3 cm in the 11L lymph node region. Furthermore, cytological diagnosis via smear examination revealed the presence of poorly differentiated cancer cells. Biopsy pathology (11 groups of lymph nodes) exhibited occurrence of atypical cells, characteristic of metastatic lung adenocarcinoma based on the medical history.

**Figure 1:**
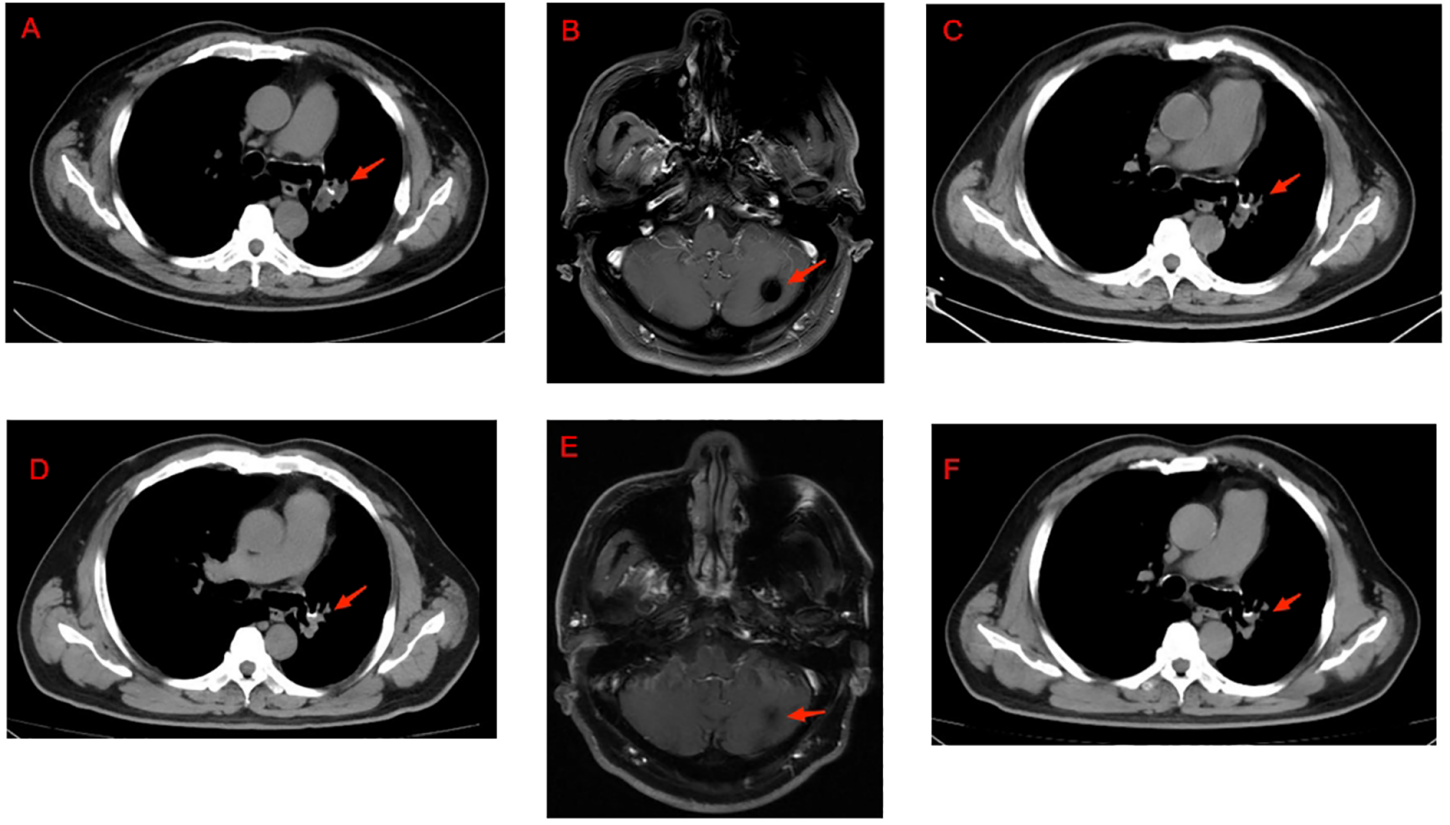
Radiological comparison before and after immunotherapy. **(A)** Lung imaging of lung cancer recurrence in October 2020. **(B)** Before receiving radiotherapy targeting the intracranial lesion. **(C)** The pulmonary lesion was reduced after four cycles of immunotherapy. **(D)** The pulmonary lesions remained stable during immunotherapy in 2022. **(E)** The intracranial lesion remained stable during immunotherapy in 2022. **(F)** The most recent lung imaging.

The patient was diagnosed with a postoperative recurrence of pulmonary adenocarcinoma (rT2aN3M1c, Stage IVB). The patient underwent stereotactic body radiation therapy (SBRT) targeting the brain metastasis, delivered as 3,797 cGy in four fractions over 7 days (**Figure 1B**).

Genetic testing was performed using next-generation sequencing (NGS, at Nanjing Geneseeq Technology, Jiangsu, China) on the tumor tissue obtained via EBUS-guided biopsy of the 11L lymph node in July 2020. The test confirmed a *BRAF* V600E mutation, with no other canonical oncogenic drivers or co-mutations (including *TP53*, *STK11*, and *KEAP1*) detected. Additionally, tumor mutational burden (TMB) analysis was performed on the archived surgical specimen from 2019, which revealed a high TMB of 13.4 mutations/Mb. Prompted by the confirmed *BRAF* V600E status, oral dabrafenib (150 mg twice daily) and trametinib (2 mg once daily) were initiated in August. During intermittent targeted therapy, the patient developed recurrent high-grade fever (peaking at 40.0 °C), nausea, vomiting, diarrhea, fatigue, myalgia, and sore throat, leading to hospitalization in the respiratory department on September 2, 2020. Cardiac biomarker analysis revealed elevated aspartate aminotransferase (AST: 293.9 U/L), lactate dehydrogenase (LDH: 2861 U/L), creatine kinase (CK: 1336 U/L), CK-MB (12.0 ng/mL), and myoglobin (591.7 ng/mL) levels, but normal troponin levels. Laboratory tests also indicated acute kidney injury (serum creatinine: 346 μmol/L), leukopenia (WBC: 2.67×10^9^/L), moderate anemia (Hb: 93 g/L), thrombocytopenia (61×10^9^/L), hypoalbuminemia, and electrolyte disturbances (hypokalemia and hyponatremia). Based on these findings, the patient was diagnosed with rhabdomyolysis, hepatorenal dysfunction, and associated complications attributed to targeted therapy. The duration of targeted therapy was less than one month. Given the severity of the adverse events, the targeted agents were permanently discontinued without attempting dose reduction or rechallenge. Supportive measures, including aggressive hydration, urine alkalinization, hepatoprotection, infection control, and electrolyte correction, resulted in symptomatic improvement and discharge. After a washout period of approximately 52 days to allow for recovery from severe toxicity, the patient was considered for immunotherapy. Subsequent follow-up demonstrated persistently elevated serum creatinine (117–145 μmol/L), consistent with progression from drug-induced irreversible acute kidney injury to chronic kidney disease (compensatory stage).

**Figure 2:**
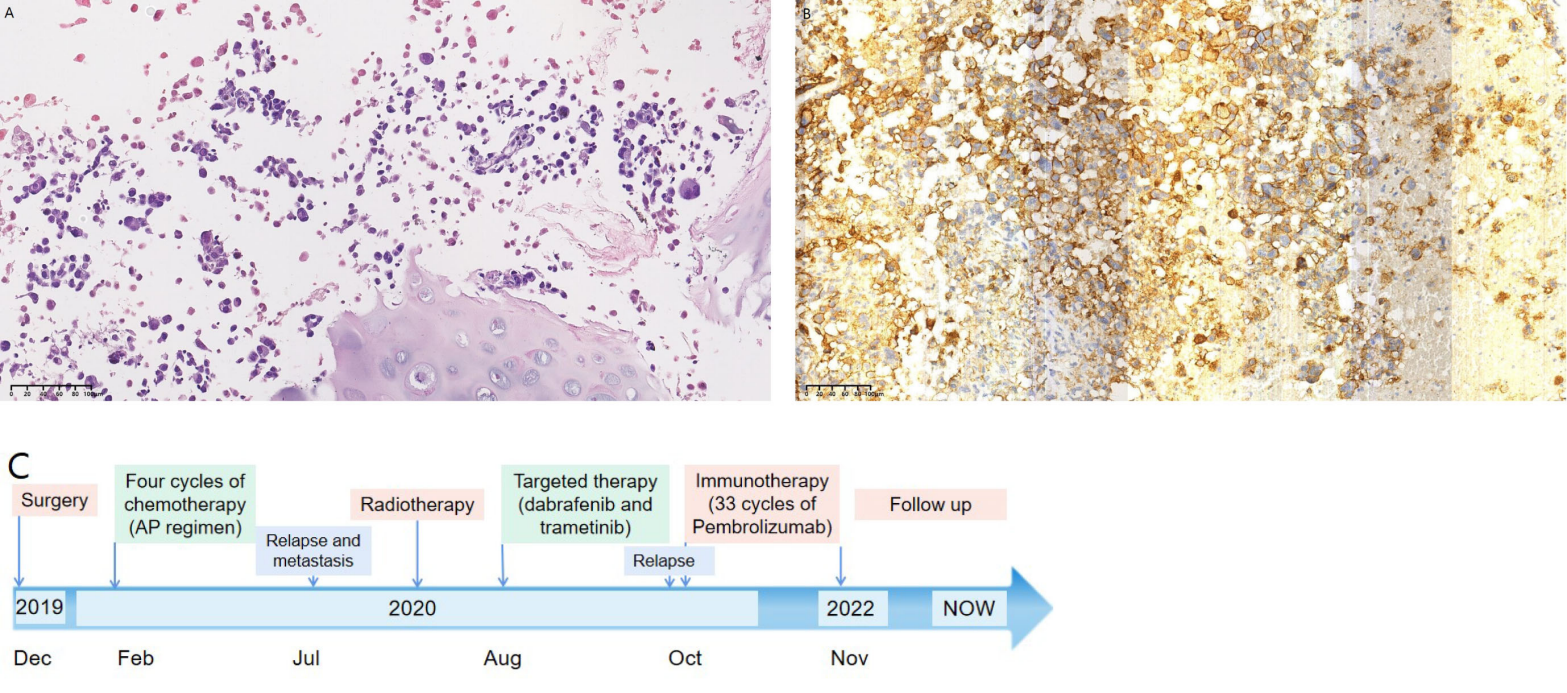
**(A, B)** Hematoxylin-eosin (HE) and immunohistochemistry staining in the lymph node. **(C)** Timeline from December 2019 to now.

In October 2020, the patient developed hoarseness. Chest CT revealed an enlarging soft tissue density with contrast enhancement in the left hilar region, suggestive of disease recurrence and left pulmonary artery invasion (**Figure 1A**). Immunohistochemical analysis of the lymph node (**Figures 2A, B**), performed in October 2020 using tissue obtained and preserved during an EBUS procedure in July 2020, suggested “PD-L1 (22C3) expression with a TPS of 90%”. Guided by this high PD-L1 expression and the patient’s intolerance to targeted therapy, on October 24, the patient was initiated on pembrolizumab monotherapy (200 mg every 3 weeks). After four cycles, repeat chest CT (**Figure 1C**) showed a significant reduction in the left para-mediastinal lesion, and brain MRI demonstrated shrinkage of the left cerebellar lesion, consistent with a partial response (PR). Immunotherapy was continued for 33 cycles until November 23, 2022. Throughout the treatment, the patient maintained stable disease without significant discomfort (**Figures 1D, E**). Throughout the 33 cycles of pembrolizumab, renal function remained stable in the compensatory stage (serum creatinine 117–145 μmol/L), with no evidence of further immune-related nephrotoxicity, allowing for the maintenance of standard dosing without adjustment. Only one instance of thyroid dysfunction (low T3) was witnessed in August 2022, and no other adverse reactions recurred.

In December 2022, the patient was diagnosed with severe COVID-19 pneumonia and achieved complete recovery following active treatment. Follow-up chest CT scans demonstrated no significant pulmonary fibrotic changes. Subsequent radiographic evaluations during the 3–6-month follow-up visits confirmed disease stability without progression (**Figure 1F**). At the time of writing this article, the patient remains alive with no evidence of disease progression (**Figure 2C**).

The patient reported that the adverse effects experienced during the brief course of targeted therapy were physically and psychologically debilitating. However, upon switching to pembrolizumab, he noted a dramatic improvement in his quality of life with no perceptible side effects. He expressed high satisfaction with the treatment outcome, which has allowed him to maintain a normal lifestyle for over four years.

## 3 Discussions

### 3.1 Monitoring adverse reactions to targeted therapies

The clinical implementation of molecularly targeted therapies has significantly improved survival outcomes for patients with non-small cell lung cancer (NSCLC) (3). Both national and international guidelines recommend dabrafenib in combination with trametinib as a first-line treatment for NSCLC harboring the *BRAF* V600E mutation. However, the therapeutic benefits of this dual-targeted regimen are accompanied by a spectrum of toxicities and adverse effects that require careful clinical management. In a phase 2 clinical trial conducted by Planchard et al., dabrafenib combined with trametinib demonstrated an overall response rate of 64% in patients with *BRAF* V600E-mutant NSCLC, although grade 3-4 adverse events were reported in 69% of participants (4). Common treatment-related toxicities include cutaneous reactions such as rash and photosensitivity, which typically require dose modification or temporary treatment interruption, as well as systemic adverse effects including hypertension, pyrexia, and elevated liver enzymes (5–7).

For severe drug-induced adverse reactions such as high-grade fever, rhabdomyolysis, and hepatic or renal impairment, no specific antidotes exist, and management primarily relies on dose reduction or treatment discontinuation. Previous reports have indicated that dose reduction of dabrafenib and trametinib can effectively prevent the occurrence of rhabdomyolysis (8). Nevertheless, the potential impact of such dose modifications on treatment efficacy remains uncertain, as conducting robust real-world studies to correlate dose adjustments with clinical outcomes in oncology patients presents significant methodological challenges.

When targeted therapy requires discontinuation due to intolerable toxicity, alternative treatment strategies must be promptly explored to maintain disease control. In the present case, the development of multiple severe adverse events attributed to dual-targeted therapy necessitated treatment cessation and transition to an alternative therapeutic approach.

### 3.2 Mechanisms underlying the durable response

The patient’s markedly elevated PD-L1 TPS of 90% (assessed with Dako 22C3 antibody) strongly predicted a favorable response to immune checkpoint inhibition. Notably, longitudinal assessment revealed an increase in PD-L1 TPS from ≥60% in the primary tumor to 90% in the post-chemotherapy metastatic lymph node. This temporal evolution mirrors recent findings from Di Federico et al. (9), who reported that PD-L1 expression significantly increases in locoregional recurrences compared to baseline (P = 0.04). In the present case, this upregulation—possibly induced by therapeutic pressure or clonal evolution—likely contributed to the exceptional therapeutic efficacy of pembrolizumab. This is corroborated by pivotal Phase III trials (KEYNOTE-024/042), which established the superiority of pembrolizumab over chemotherapy in advanced NSCLC with PD-L1 TPS ≥1%, with the most pronounced benefit observed in patients exhibiting high PD-L1 expression (TPS ≥50%) (10, 11).

Furthermore, the patient’s 30-year smoking history represented another crucial positive predictive factor. Extensive retrospective analyses indicate that smokers, particularly heavy smokers, exhibit higher response rates to immunotherapy, which is likely attributable to the high TMB induced by tobacco carcinogens. Consistent with this association, retrospective NGS analysis of the patient’s surgical specimen confirmed a high TMB of 13.4 mutations/Mb. Although this assessment was performed on the archival surgical specimen from 2019, a recent large-scale study demonstrated high intrapatient concordance of TMB values (Spearman’s ρ = 0.80) between samples collected at different times, confirming that TMB remains a stable biomarker over the disease course (9). Importantly, emerging evidence indicates that TMB and PD-L1 are independent predictive biomarkers. Patients with tumors characterized by both high TMB and high PD-L1 expression (TPS ≥50%)—so-called “dual-positive” tumors—derive the greatest survival benefit from immunotherapy. In a pivotal study, this population achieved an objective response rate of 57%, which was significantly superior to that of patients with only one or neither marker elevated (12). Elevated TMB generates more neoantigens, enhancing immune recognition and antitumor activity (13). Moreover, the neoantigen-rich environment in smokers facilitates the infiltration of effector T cells, which secrete interferon-γ and subsequently induce PD-L1 upregulation as an adaptive immune resistance mechanism (14). Clinical evidence supports that smokers with lung adenocarcinoma show significantly higher rates of PD-L1 TPS ≥50% compared to never-smokers (15), and among patients with BRAF-mutant NSCLC, smokers demonstrate significantly longer progression-free survival (PFS) than non-smokers (16).

The role of the *BRAF* V600E mutation in this context is multifaceted. Although *BRAF*-mutant NSCLC overall is associated with high PD-L1 expression and positivity rates (17–19), the relationship between mutation subtype, smoking, and PD-L1 levels is complex. While the V600E subtype occurs in both smokers and non-smokers, non-V600E *BRAF* mutations are more strongly linked to smoking history (18, 20). In this case, while the *BRAF* mutation functioned as the primary oncogenic driver, the durable clinical control was likely driven by the synergistic convergence of exceptionally high PD-L1 expression, confirmed high TMB, and smoking-related immunogenicity.

### 3.3 Considerations for molecular testing strategies

In this case, NGS was employed for genetic testing. Regarding *BRAF* mutation testing, according to a recent expert consensus (21), the commonly used methods have distinct characteristics: qPCR (Quantitative real time polymerase chain reaction) offers high sensitivity (approximately 0.001%), rapid turnaround, and cost-effectiveness, making it the preferred routine method; immunohistochemistry with anti-*BRAF* V600E antibody shows high specificity for the V600E mutation and is suitable for rapid screening but cannot detect non-V600E mutations; NGS provides comprehensive detection of various alterations including point mutations and fusions, with high sensitivity, and is considered the gold standard for obtaining a complete molecular profile, though it requires higher cost and bioinformatic expertise. As highlighted in the aforementioned consensus, NGS enables the detection of a broader spectrum of *BRAF* mutations (such as fusions and non-V600 point mutations) and co-mutations, which is crucial for formulating precision treatment plans. For patients with complex treatment responses as illustrated here, or when standard testing fails to identify a driver gene, employing more comprehensive analytical approaches such as whole-exome sequencing (WES) or whole-genome sequencing (WGS) could help uncover rare variants and explore immunotherapy-related biomarkers like neoantigen load, thereby providing a more thorough basis for in-depth precision therapy.

### 3.4 Discussion of other potential mechanisms for immunotherapy success

Beyond the well-established predictive value of high PD-L1 expression and significant smoking history, and high TMB, other factors specific to this case’s clinical course may have contributed to the exceptional response to immunotherapy.

First, the potential role of SBRT for brain metastasis in priming a systemic immune response warrants consideration. Radiotherapy can induce immunogenic cell death, releasing tumor neoantigens and danger signals that may function as an *in situ* vaccine. This process can promote the activation and expansion of tumor-specific T cells. When followed by immune checkpoint inhibition, this may lead to an abscopal effect—where localized radiation treatment elicits a systemic anti-tumor immune response (22, 23). This systemic potentiation is supported by a pooled analysis of randomized trials, which demonstrated that adding radiotherapy (including SBRT) to pembrolizumab significantly improved abscopal response rates and survival in metastatic NSCLC (24). Crucially, the high TMB (13.4 muts/Mb) detected in this patient likely amplified this effect. High-TMB tumors, which are more immunogenic and prevalent in certain subtypes like NSCLC, are theoretically more likely to mount a robust systemic response following such *in situ* vaccination (25). Chae et al. demonstrated that in patients with NSCLC treated with immune checkpoint blockade, higher TMB is significantly associated with prolonged overall survival, likely due to an increased neoantigen load that facilitates a more robust anti-tumor immune response (26). Furthermore, emerging evidence suggests that a high TMB may itself be a biomarker for increased sensitivity to radiotherapy, predicting better local control after radiation (27). In this patient, the SBRT administered to the cerebellar lesion prior to starting pembrolizumab may have helped create a more favorable immune environment, potentially enhancing the subsequent efficacy of immunotherapy against the systemic disease. A study (28) demonstrated that radiotherapy with a CTLA-4 antibody induces T-cell exhaustion, whereas the addition of a PD-L1 antibody can reverse this effect and improve the response rate of melanoma to this combination therapy. However, radiotherapy is inherently a localized treatment modality; even though it can elicit an immune-mediated “abscopal effect,” only a minority of patients ultimately benefit from this phenomenon (29). The presence of a high TMB in this patient may have been a key factor that predisposed her to be among this benefiting minority, by providing the essential neoantigenic substrate for the SBRT-primed systemic immune response.

Second, the interplay between the *BRAF* V600E mutation and the tumor immune microenvironment merits discussion. While *BRAF* V600E is a potent oncogenic driver, its presence does not preclude response to immunotherapy. This case demonstrates that a high PD-L1 level can serve as a dominant predictive biomarker, even in the context of a *BRAF* V600E mutation. The patient’s robust response suggests that the *BRAF* V600E mutation did not create an inherently immunosuppressive “immune-desert” phenotype in this instance. Instead, the tumor remained immunologically responsive, as evidenced by the high PD-L1 expression, which likely acted as a key mechanism of adaptive immune resistance. The success of immunotherapy here was likely driven by the potent combination of a targetable immune checkpoint (PD-L1) and a pre-existing, albeit inhibited, T-cell response, which may have been further amplified by the prior radiotherapy.

### 3.5 Identifying predictors of good prognosis (PFS > 48 months)

Immune-related adverse events (irAEs) associated with immune-targeted inhibitors are randomized and multisystemic. Most patients experience adverse events with PD-I/PD-L1 inhibitors; however, adverse events that are severe enough to reach grade 5 occur in a minority of patients. A meta-analysis of 3,450 patients in a total of 7 randomized controlled trials (RCTs), including NSCLC (4 trials) and melanoma (3 trials), proposed that compared to chemotherapy, PD1/PD-L1 inhibitors are associated with a lower risk of treatment-related symptoms (fatigue, anorexia, nausea, diarrhea, constipation, and sensorineural neuropathy), but a higher risk for immune-related adverse events (rash, pruritus, colitis, elevated aminotransferases, thyroid diseases, and full- and high-grade pneumonia) (30).

Notably, the occurrence of grade 1–2 irAEs has been linked to improved clinical outcomes. Better prognosis in patients with late-onset irAEs may correlate with an extended treatment duration before discontinuation, suggesting that milder and delayed toxicity following pembrolizumab monotherapy predicts superior clinical outcomes and prognosis. This observation reinforces the rationale for immunotherapy, prolongs the duration of immunosuppression, and enables a longer treatment maintenance (31). Importantly, the 5-year overall survival (OS) rate exceeded 25% in patients with a PD-L1 TPS ≥50%. Pembrolizumab demonstrates a tolerable long-term safety profile, with minimal delayed or *de novo* toxicity (32).

These findings are supported by those of Reck et al. in the KEYNOTE-024 trial. The study demonstrated that, in patients with NSCLC exhibiting PD-L1 TPS ≥50%, first-line pembrolizumab monotherapy provided clinically meaningful improvements in OS, PFS, objective response rate, and duration of response compared to platinum-based chemotherapy. Furthermore, among the 102 patients who completed 35 cycles of pembrolizumab monotherapy, the 4-year OS rate was 61.8%, with 41 patients (40.2%) surviving without disease progression (PD) or requiring subsequent therapy. Notably, among the 33 patients who developed PD after completing 35 cycles and received a second course of treatment, the median survival time was 63.7 months (range: 52.0–75.2). Within this subgroup, 5 patients (15.2%) achieved partial response and 20 (60.6%) maintained stable disease, yielding a disease control rate of 75.8%. Additionally, two patients (6.1%) remained alive without PD or further therapeutic intervention. These data underscore the long-term clinical benefits of pembrolizumab in patients with advanced NSCLC with high PD-L1 expression (33).

## 4 Conclusion

For patients with *BRAF* V600E-mutated NSCLC who demonstrate intolerance to targeted therapies, immunotherapy may offer a viable salvage strategy. In this case, the durable response to pembrolizumab was likely driven by a favorable convergence of predictive biomarkers, including high PD-L1 expression, high TMB, and a significant smoking history. While this report highlights a potential alternative therapeutic approach, these findings are based on a single case and should be validated through further clinical investigation to refine precision treatment paradigms for this specific patient subgroup.

## Data Availability

The original contributions presented in the study are included in the article/supplementary material. Further inquiries can be directed to the corresponding author.
